# Supplementary material for: Comparative Study of the Role of Interepithelial Mucosal Mast Cells in the Context of Intestinal Adenoma-Carcinoma Progression
Source: Cancers (Basel). 2022 Apr 30;14(9):2248. doi: 10.3390/cancers14092248 (PMC9105816; doi:10.3390/cancers14092248)
Supplement: Supplementary file 1 [file cancers-14-02248-s001.zip › cancers-1678186 - supplementary Figrue S1-S9.pdf]

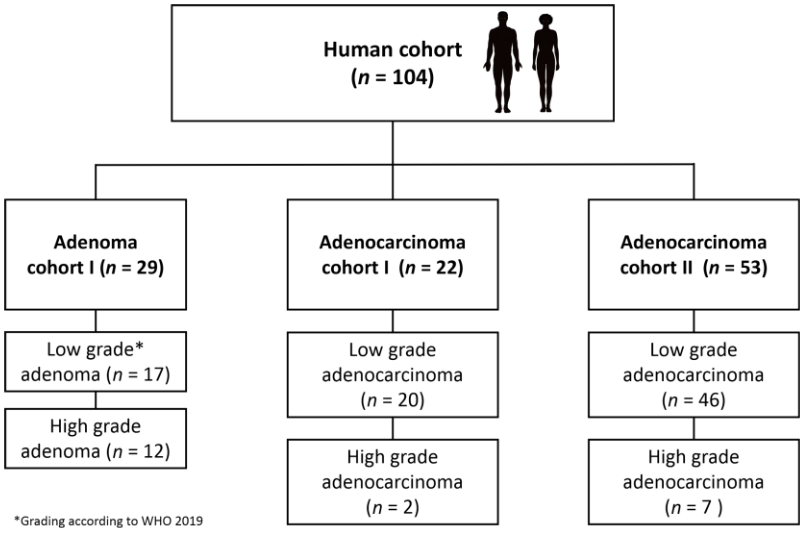

Figure S1. Overview of the human cohort (*n* = 104). Cohort I of *n* = 17 low-grade and *n* = 12 high-grade adenomas and *n* = 22 adenocarcinomas was extended by adenocarcinoma cohort II (*n* = 53). Grading of adenomas and carcinomas (low-grade; high-grade) was accomplished according to the current human WHO classification [46].

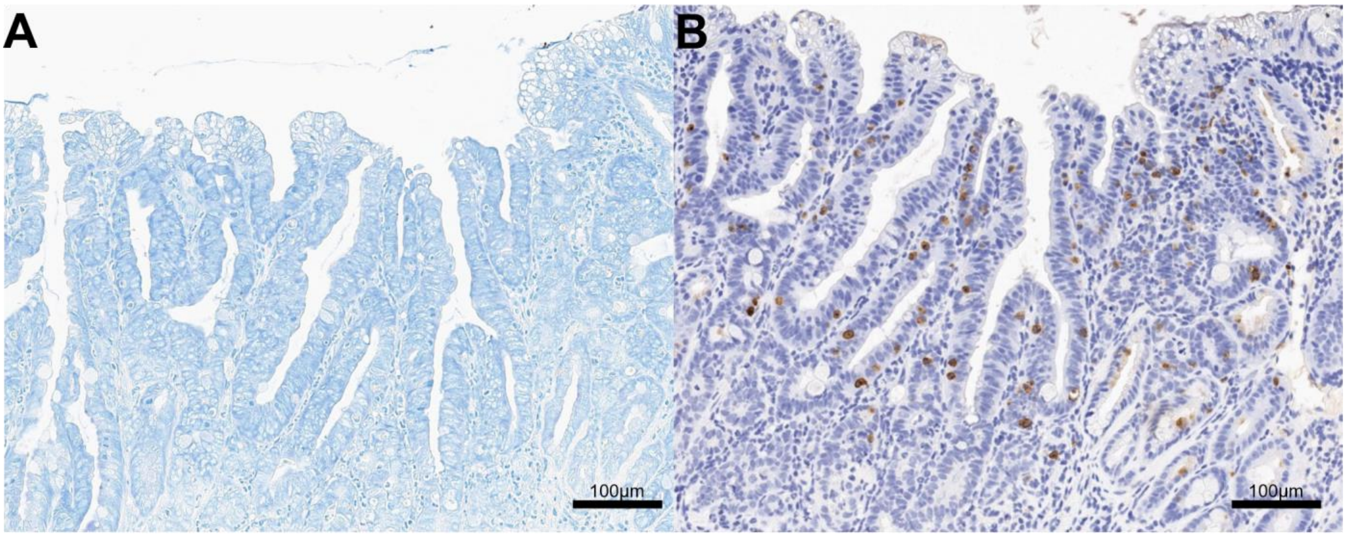

Figure S2. A,B: Interepithelial mucosal mast cells (ieMMCs) in the intestinal mucosa of mice were not identifiable by metachromatic staining (toluidine blue). Serial sections for toluidine blue (A) and MCPT1 (B).

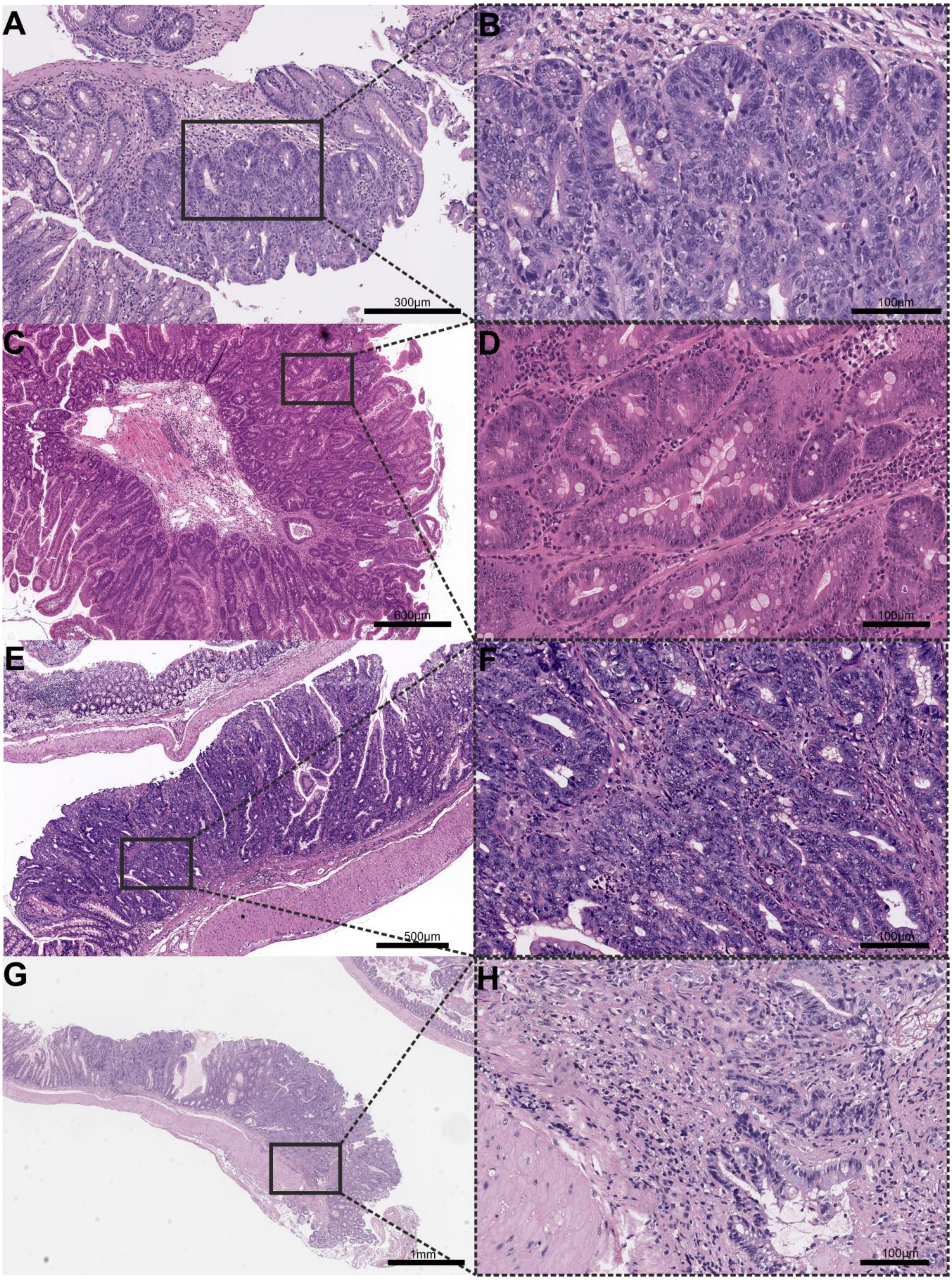

Figure S3. Grading of murine lesions was performed according to Nolte et al., 2016 (Table S4) [45]. A–H: Murine lesions were graded as atypical hyperplasia (A,B); low-grade adenoma (C,D); high-grade adenoma (E,F); or invasive carcinoma (G,H).

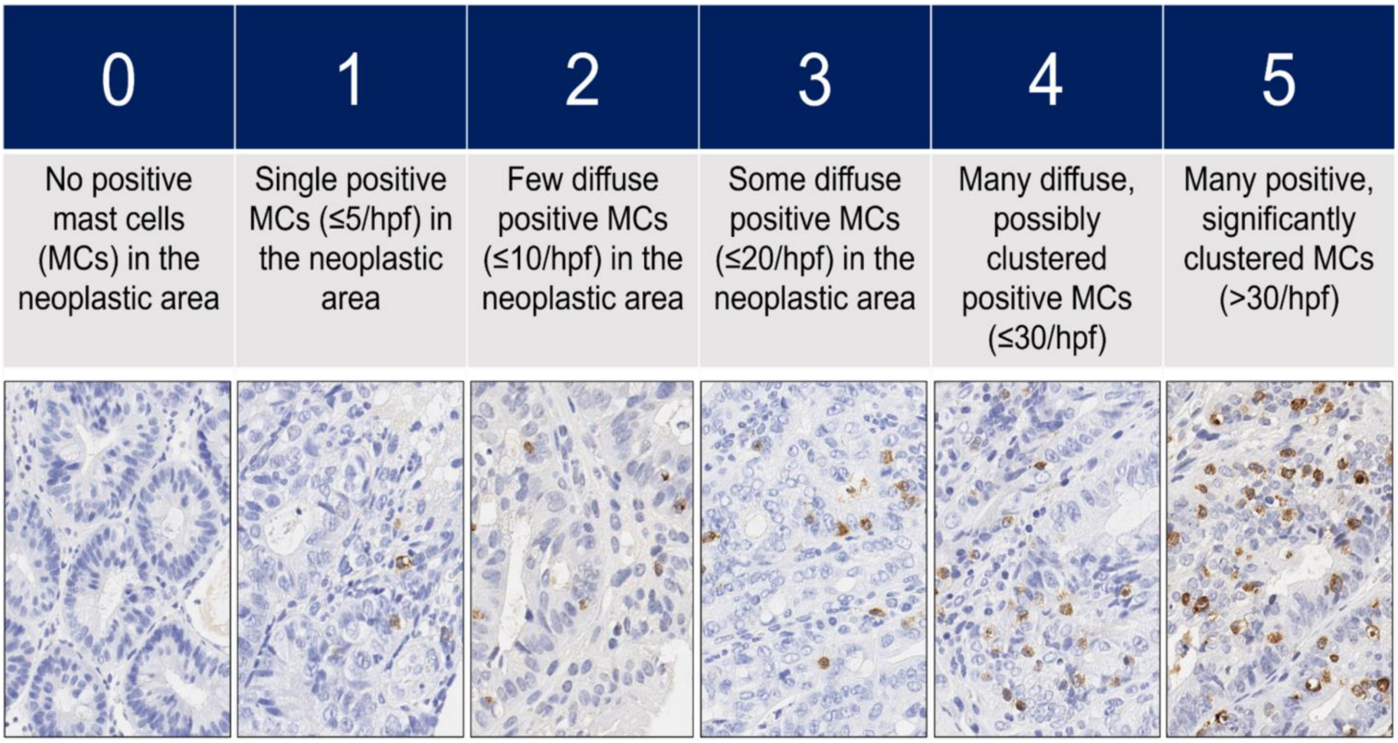

Figure S4: The employed semiquantitative score (0 to 5) for mucosal mast cells (ieMMCs and lpMMCs) in hotspots of murine intestinal lesions. Corresponding to Table 1 (hpf: high-power field, 40×).

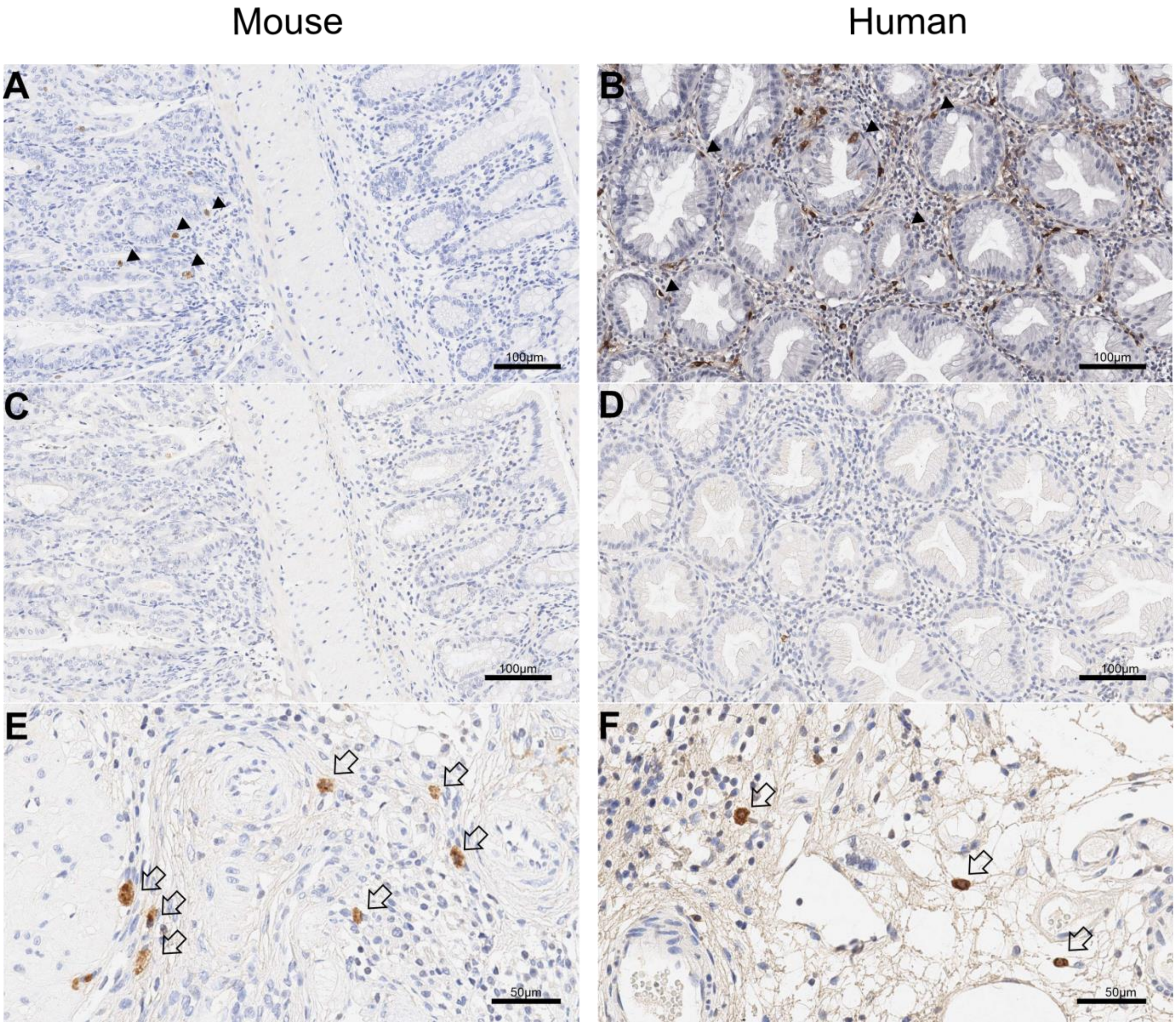

Figure S5: A–F: IeMMCs (A,B) were negative for histamine (C,D), while connective tissue mast cells (CTMCs) of the intestine were positive for histamine (E,F). A,C: Murine high-grade adenoma with MPCT1^{+} ieMMCs (arrowheads) (A), which were negative for histamine in the serial section (C). B,D: Human low-grade sessile serrated adenoma with MCT^{+} ieMMCs (arrowheads) (B), which were negative for histamine in the serial section (D). E,F: CTMCs in the intestine of mice (E) and humans (F) were positive for histamine (arrows).

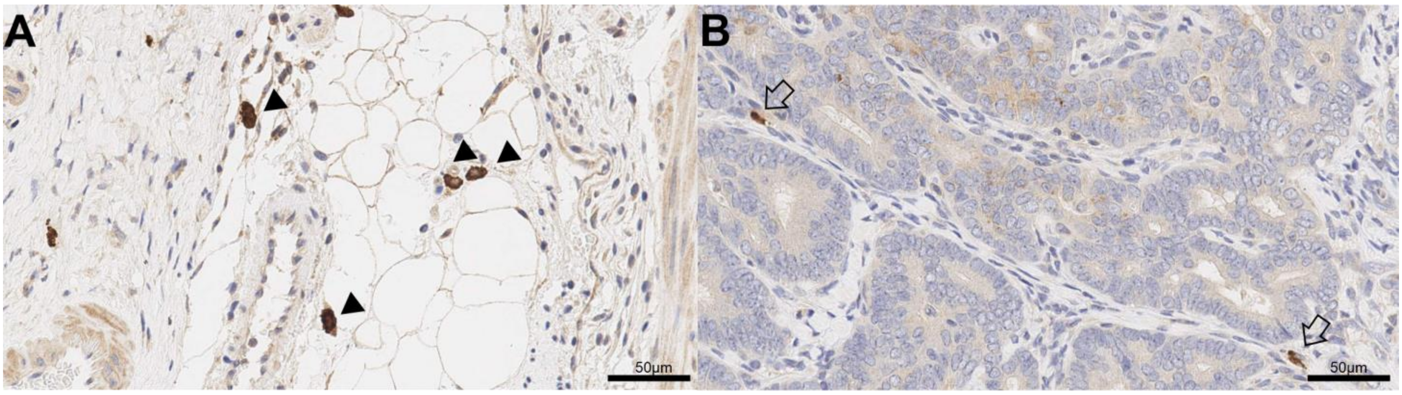

Figure S6: A: Murine connective tissue mast cells (CMTCs) were MCPT6^{+} (MC Tryptase^{+}) and appeared densely granulated and round (arrowheads). B: Lamina proprial mucosal mast cells (lpMMCs, MCPT6^{+}) of mice were generally slender and smaller than CTMCs (arrows).

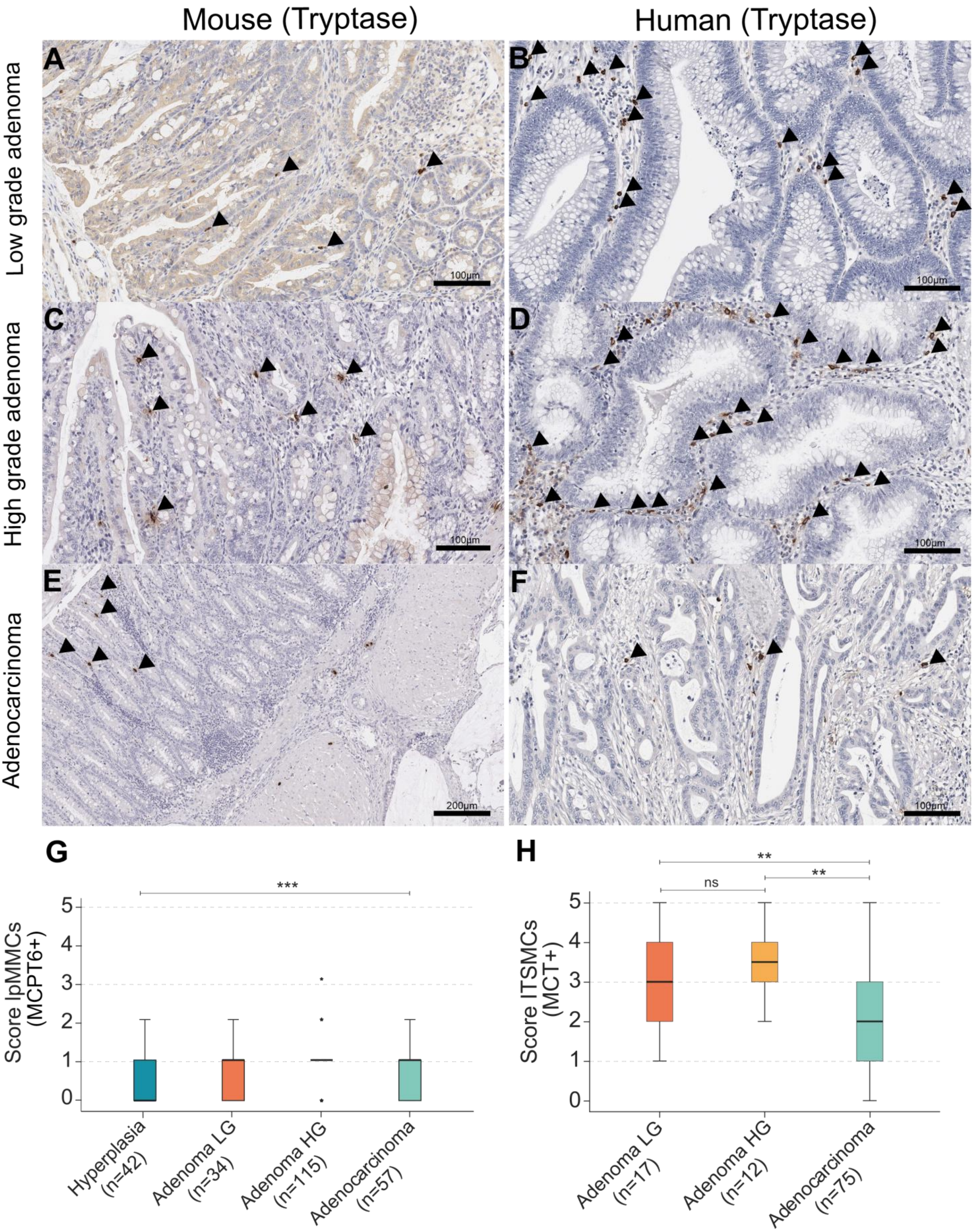

Figure S7: A–F: LpMMCs in the adenoma-carcinoma sequence of mouse (A,C,E) and intratumoral stromal MCs (ITSMCs) in the human adenoma-carcinoma sequence (B,D,F). G: Scores of lpMMCs were lower in murine hyperplastic precursor lesions than in adenomas and carcinomas (Kruskal–Wallis test, $p = 0.001$). H: Contradictorily, stromal MC scores were higher in human precursor lesions than in carcinomas (Kruskal–Wallis test, $p < 0.001$).

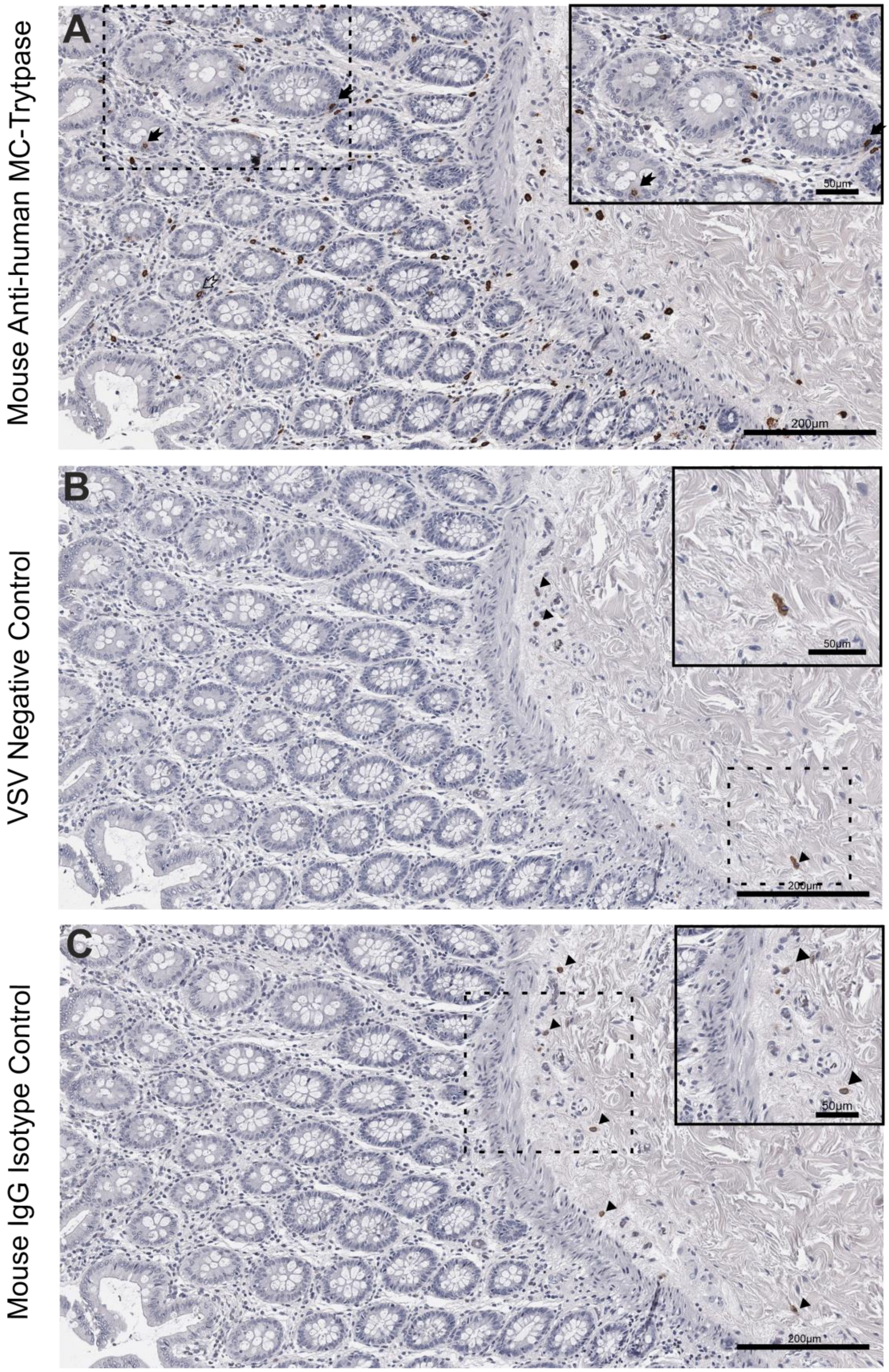

Figure S8: A–C: Individual human intratumoral stromal mast cells (ITSMCs) appeared to be nonspecifically positive due to heparin presence as previously described [52]. A,B: MC tryptase-positive (MCT^{+}) ieMMCs (A) (arrows) were negative in vesicular stomatitis virus (VSV) IHC (B) and in mouse isotype IgG control (C), serving as negative IHC controls. B,C: Connective tissue mast cells (CTMCs) were occasionally positive in negative controls. Thus, MCT IHC (A) was a specific marker for human intestinal ieMMCs.

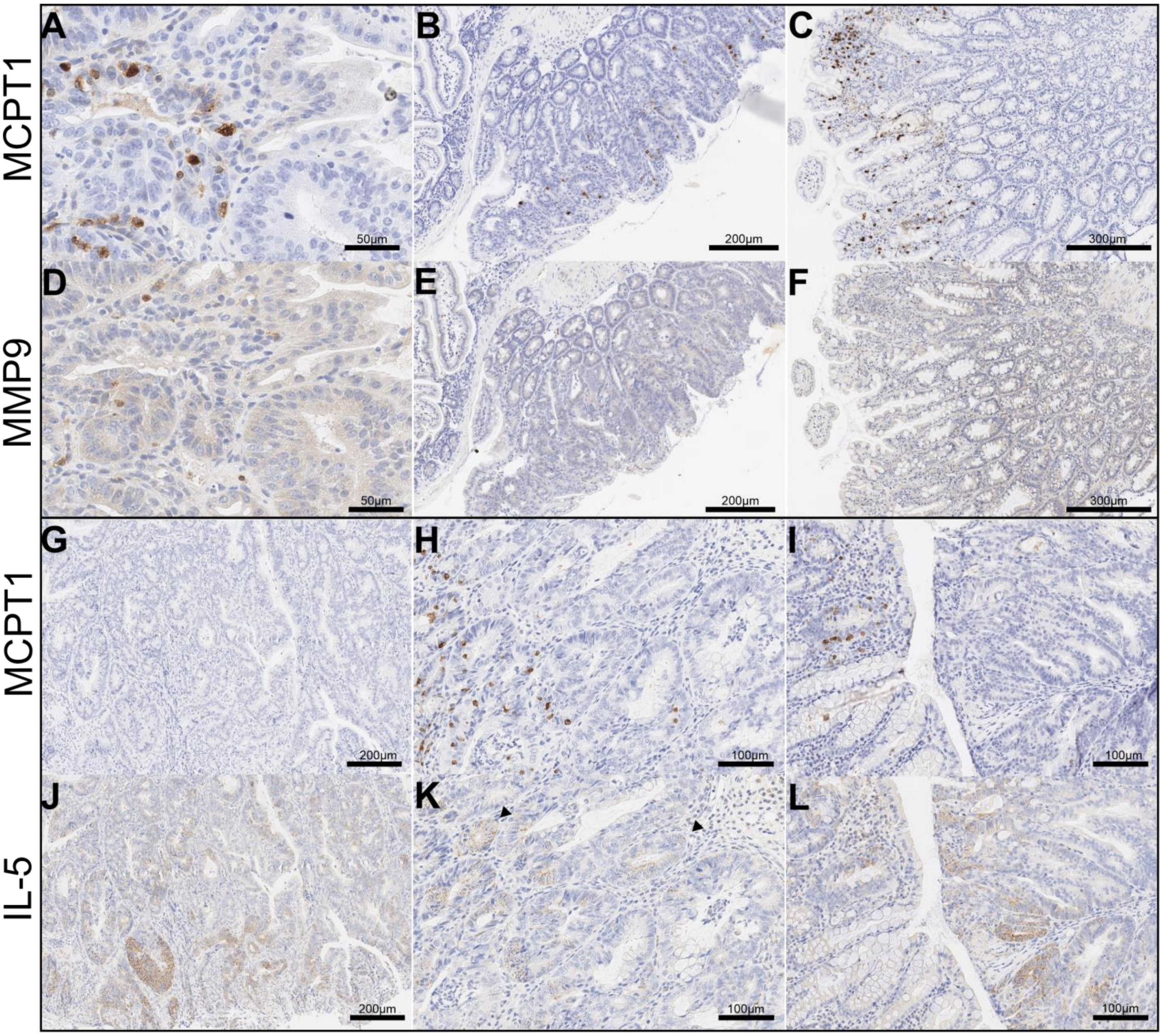

Figure S9: A–F: MCPT1⁺ ieMMCs (A–C) were not coincident with MMP9⁺ cells (D–F) in serial sections. G–L: Absence (G) or presence of MCPT1⁺ ieMMCs in murine intestinal neoplasia. J–L: Luminal intestinal crypt cells were more often positive for IL-5 than basal crypt cells (J–L), but IL-5 positivity did not clearly correlate with ieMMC density (arrowheads).
